# Spatial modeling of HIV prevalence in Malawi using generalized additive models

**DOI:** 10.3389/fepid.2026.1867778

**Published:** 2026-07-08

**Authors:** Zacharie Tsala Dimbuene, Crispin Mabika Mabika, Blandine Bawawana Bavwidinsi, Emmanuel Juakaly Wayisovia, Hugues Sampasa-Kanyinga

**Affiliations:** 1 School of Population and Development Sciences, University of Kinshasa, Kinshasa, Democratic Republic of Congo; 2 Faculty of Economics and Management, University of Kinshasa, Kinshasa, Democratic Republic of Congo; 3 Faculty of Economics and Management, Official University of Semuliki, Beni, Democratic Republic of Congo; 4 Union for African Population Studies, Accra, Ghana; 5 Observartoire Français des Conjonctures Economiques (OFCE), Paris, France; 6 Faculty of Medicine, University of Kinshasa, Kinshasa, Democratic Republic of Congo

**Keywords:** generalized additive model, HIV, Malawi, shapley value, thin-plate spline

## Abstract

**Introduction:**

Malawi has made substantial progress in HIV prevention and treatment, yet HIV prevalence remains unevenly distributed across the country. Sub-national estimates are needed to guide targeted interventions.

**Methods:**

We analyzed individual-level HIV biomarker data from the 2016 Malawi Demographic and Health Survey. A spatial modeling approach was applied to capture broad geographic patterns alongside sociodemographic determinants. High-resolution maps of predicted HIV prevalence were generated to visualize fine-scale differences across districts.

**Results:**

The analysis revealed persistent geographic disparities, with the highest prevalence concentrated in southern Malawi and more varied patterns in central and northern regions. Although geography contributed to explaining HIV variation, sociodemographic factors—including age, education, sex, and household characteristics—were the primary drivers in most districts. Geography emerged as the leading contributor in only 18% of areas.

**Discussion and Conclusion:**

These findings provide policy-relevant, sub-national evidence to support more precise targeting of HIV prevention, testing, and treatment efforts. They underscore the importance of tailoring interventions to both geographic and sociodemographic contexts to accelerate progress toward epidemic control.

## Introduction

Malawi continues to experience one of the highest human immunodeficiency virus (HIV) burdens in eastern and southern Africa, despite substantial progress in expanding prevention and treatment services (1–3). Recent national estimates indicate that approximately one million people are living with HIV, with prevalence remaining above 6% among adults aged 15–64 years. The country has made notable strides toward achieving the Joint United Nations Program on HIV/AIDS (UNAIDS) 95–95–95 targets, supported by large-scale investments in testing, antiretroviral therapy (ART), and prevention programs (4–6). UNAIDS 95 95–95 is the Joint United Nations Programme on HIV/AIDS (UNAIDS) set of ambitious targets to enable 95% of people living with HIV to know their serological status, 95% of people diagnosed as HIV-positive to receive continuous antiretroviral therapy and 95% of people on antiretroviral therapy to have viral suppression by 2025. Population-based HIV Impact Assessments (PHIAs) conducted in 2015–16 and 2020–21 demonstrated declines in incidence and improvements in viral suppression; however, they also revealed persistent geographic disparities in HIV burden across districts and regions^7^. These spatial inequalities underscore the need for analytic approaches capable of disentangling the complex interplay between individual-level determinants and place-based factors that shape HIV risk (7–9).

Previous spatial analyses of HIV prevalence in Malawi have relied heavily on model-based geostatistics, kernel density estimation, or Bayesian small-area estimation frameworks such as the Naomi model (2, 7, 8, 10, 11). While these approaches have advanced understanding of sub-national heterogeneity, they often face important methodological limitations. Many models assume stationarity or impose strong parametric structures that may obscure nonlinear relationships between predictors and HIV outcomes. Others focus primarily on estimating prevalence or incidence without explicitly quantifying the relative contributions of individual-level covariates vs. unmeasured spatial processes (12). As a result, prior studies have struggled to separate the effects of population composition from those of geographic context, limiting their ability to determine whether observed spatial patterns reflect who lives in an area or the characteristics of the area itself. Moreover, traditional spatial smoothing methods may mask local variation or fail to provide interpretable measures of variable importance, thereby reducing their utility for targeted intervention planning.

To address these gaps, the present study integrates generalized additive models (GAMs) with Shapley value decomposition to produce a novel and interpretable mapping of HIV prevalence across Malawi. GAMs allow flexible modeling of nonlinear relationships between HIV risk and demographic, socioeconomic, and behavioral predictors, while simultaneously capturing smooth spatial trends (13, 14). Shapley decomposition—adapted from cooperative game theory—provides an additive, model-agnostic framework for quantifying the contribution of each predictor, including spatial location, to the model's predictions (15, 16).

By combining these methods and applying thin-plate spline smoothing to the resulting Shapley surfaces, this study generates high-resolution maps that distinguish the influence of individual-level characteristics from underlying geographic structure. This approach offers a more transparent and nuanced understanding of HIV risk heterogeneity, enabling clearer identification of areas where prevention strategies should prioritize population-level interventions vs. place-based structural responses. In doing so, it advances both methodological rigor and practical relevance for HIV epidemic control in Malawi.

## Methods

### Study setting

Malawi is a landlocked country in southeastern Africa, characterized by substantial geographic, socioeconomic, and health-system diversity. For more details, see (12). With a predominantly rural population and marked regional disparities in access to healthcare, water, sanitation, and maternal services, the country faces persistent challenges in reducing HIV. Administratively divided into three regions and 28 districts, Malawi's health landscape reflects a combination of structural constraints and localized vulnerabilities that contribute to HIV inequalities. These contextual features make Malawi an informative setting for examining spatial heterogeneity in HIV and for assessing how geographic context shapes model-based predictions.

### Data sources and variables measurements

#### Data sources

The present study used data from the 2015–2016 Malawi Demographic and Health Survey (hereafter, 2016 MDHS). The 2016 MDHS is a cross-sectional nationally representative survey (freely available at DHS Program) which collected information on households, women and men of reproductive age, anthropometric measures, contraception and family planning, and other topics. Administrative boundaries were obtained from the R package rgeoboundaries (17). For the current study, level-2 administrative boundaries were used to map HIV prevalence.

#### Variables measurements

##### Outcome variable

The biomarker 2016 MDHS contains information about HIV status. The original categorical variable about HIV test was coded 0 “negative”, 1 “positive”, 7 “indeterminate”, and 9 “inconclusive”. Inconclusive and indeterminate cases were removed from final analyses.

##### Independent variables

Factors associated with HIV were retrieved from previous research (12, 18–22). Given the computationally-intensive approach adopted in this study (See sub-section below on *Statistical Framework*), only a few variables were included in the analyses, including respondents' age (in single years) and education (in single completed years), gender (0 = Male; 1 = Female), household wealth index (Poo*r* = 40% bottom; Middle = 20%; Rich = 40% top), urban residence (0 = rural; 1 = urban), and HIV testing (1 = Tested and received results; 2 = Tested and did not receive results; 3 = Never tested).

#### Statistical framework

##### Generalized additive model for HIV risk: theory and estimation

**Figure 1:**
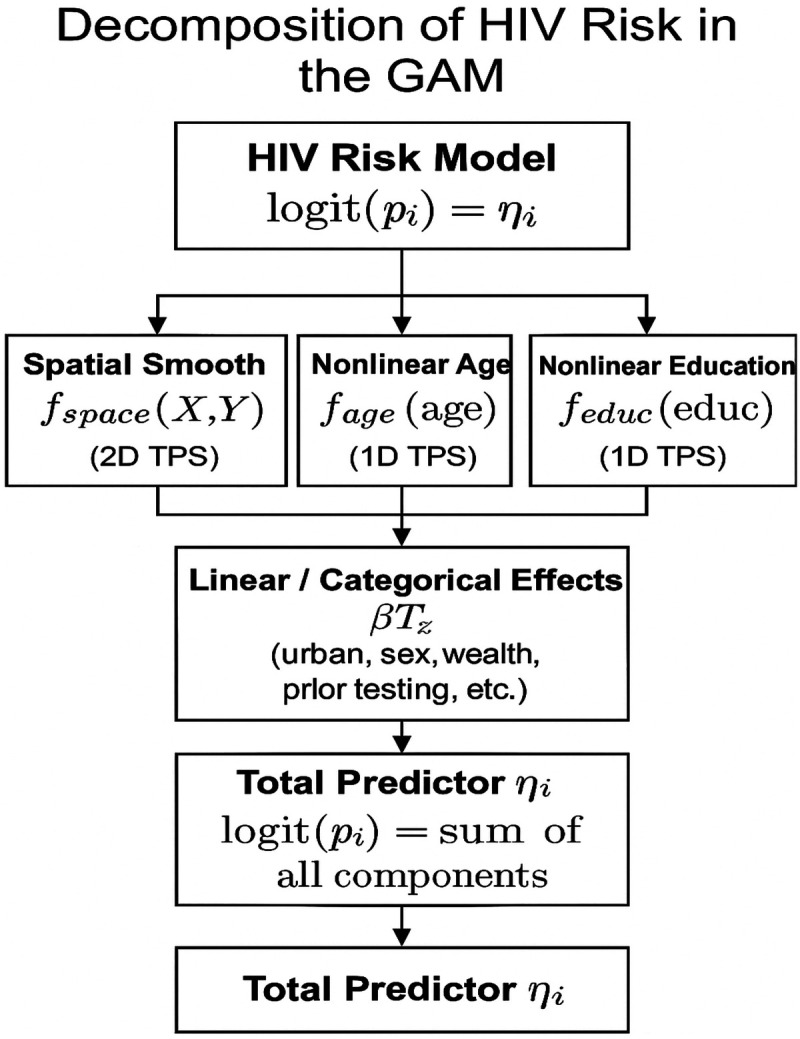
Decomposition of HIV risk in the generalized additive models (GAM).

The paper modeled the probability of HIV infection using a generalized additive model (GAM) with a binomial likelihood and logit link (See Figure 1). Let $Y_{i}$ denote the HIV serostatus of individual *i*, where $Y_{i}=1$ if the individual tested positive and $Y_{i}=0$, otherwise. Therefore, the observed number of HIV-positive cases in district *i* follows a binomial distribution with underlying prevalence $p_{i}$ as shown in Equation 1:

$$Y_{i}∼\mathrm{Binomial}(\;p_{i}),\;\mathrm{logit}(\;p_{i})=\eta_{i}$$ (1)

The linear predictor $\eta_{i}$ in Equation 2 was specified as an additive combination of smooth nonlinear functions and parametric covariate effects:

$$\eta_{i}=f_{\mathrm{space}}(X_{i},Y_{i})+f_{\mathrm{age}}(\mathrm{ag}e_{i})+f_{\mathrm{educ}}(\mathrm{edu}c_{i})+z_{i}^{⊤}\beta$$ (2)

where $\left(X_{i},Y_{i}\right)$ are geographic coordinates, $f_{\mathrm{space}}$ is a two-dimensional thin-plate spline capturing spatially structured variation in HIV risk, $f_{\mathrm{age}}$ and $f_{\mathrm{educ}}$ are univariate thin-plate splines for age and education, and $z_{i}$ contains categorical and linear predictors (e.g., urban residence, sex, household wealth index, and prior HIV testing history).

Each smooth function $f_{m}(.)$ was represented using a penalized regression spline basis in Equation 3. For a generic smooth term:

$$f_{m}(x)=\overset{K_{m}}{\underset{\;j=1}{\sum }}\theta_{mj}B_{mj}(x)$$ (3)

where $B_{mj}(.)$ are thin-plate spline basis functions and $\theta_{mj}(.)$ are coefficients. Smoothness (Equation 4) was controlled by a quadratic penalty on the curvature of the function:

$$\mathrm{penalty}(\;f_{m})=\lambda_{m}\;\theta_{m}^{⊤}S_{m}\theta_{m}$$ (4)

where $S_{m}$ is the spline penalty matrix and $\lambda_{m}$ is the smoothing parameter. The spatial smooth $f_{\mathrm{space}}(X_{i},Y_{i})$ used a two-dimensional thin-plate spline basis (Equation 5) that minimizes the bending-energy functional:

$$J(f)=\int \;\;\;\int \left[{\left(\frac{\partial^{2}f}{\partial x^{2}}\right)}^{2}+2{\left(\frac{\partial^{2}f}{\partial x\partial y}\right)}^{2}+{\left(\frac{\partial^{2}f}{\partial y^{2}}\right)}^{2}\right]\;dx\;dy$$ (5)

Model estimation proceeded by maximizing the restricted maximum likelihood (REML) criterion, which treats spline coefficients as Gaussian random effects and jointly estimates the smoothing parameters $\lambda_{m}$ and the regression coefficients β. This approach provides stable smoothing parameter selection and helps guard against overfitting. The resulting model decomposes HIV risk into spatially structured variation, nonlinear demographic effects, and linear or categorical covariate contributions.

#### Spatial smoothing of shapley contributions

To characterize large-scale spatial patterns in model behavior, the paper treated the observation-level Shapley contributions for the spatial component of the model as point samples of an underlying continuous spatial field. Let $\left(x_{i},y_{i}\right)$ denote the geographic coordinates of observation *i*, and let $z_{i}=\phi_{i,\mathrm{space}}$ represent the corresponding Shapley contribution. Although Shapley values are defined at the individual level, the spatial component often reflects latent geographic structure (e.g., unmeasured environmental exposures, health-system access, or spatially structured risk factors). We therefore estimated a smooth function f(x,y) such that $f(x_{i},y_{i})\approx z_{i}$, using thin-plate spline (TPS) regression (Equation 6).

TPS smoothing solves the penalized least-squares problem:

$$\underset{f}{\min}\left\{\overset{n}{\underset{i=1}{\sum }}{\left(z_{i}-f(x_{i},y_{i})\right)}^{2}\;+\;\lambda \;J(f)\right\},$$ (6)

where J(f) is the bending-energy functional, as shown in Equation 5. This formulation yields the smoothest possible surface that remains consistent with the observed Shapley contributions. The solution takes the form of a radial basis expansion with the kernel $\phi (r)=r^{2}\log\;r$, ensuring global smoothness and stable interpolation across irregularly spaced points. Predictions from the fitted TPS model were evaluated on a regular grid spanning the study area and subsequently masked to the administrative boundaries to produce a spatially coherent surface of estimated Shapley contributions.

Because TPS smoothing is a nonlinear transformation of the original Shapley values, the resulting surface does not satisfy the Shapley efficiency or additivity axioms. Specifically, $\hat{\;f}(x_{i},y_{i})$ does not necessarily equal the original $\phi_{i,\mathrm{space}}$, and the smoothed surface cannot be summed with other feature-level Shapley components to reconstruct the model prediction. Accordingly, the TPS surface should be interpreted as a nonparametric estimate of the underlying spatial structure in the model's behavior, rather than as a replacement for the exact Shapley values.

##### Limitations: interpretation of smoothed shapley surfaces

Although smoothing Shapley values provides a coherent visualization of spatial structure, it introduces several methodological limitations. First, TPS smoothing is a nonlinear operator; therefore, the resulting surface no longer satisfies the Shapley efficiency or additivity axioms. The smoothed values cannot be summed with other feature contributions to recover the model prediction, and they should not be interpreted as exact Shapley values at unobserved locations. Second, smoothing attenuates local variability and may obscure fine-scale heterogeneity present in the raw Shapley contributions. Third, the TPS surface represents an estimate of a latent spatial trend rather than a causal spatial effect; it reflects the model's internal structure and the spatial distribution of covariates, not necessarily underlying epidemiological processes. Finally, the choice of smoothing parameter and grid resolution may influence the appearance of spatial patterns, although large-scale gradients are generally robust. These limitations do not undermine the utility of the smoothed surfaces for interpretation; rather, they clarify that the maps are best viewed as descriptive summaries of spatial structure in the model's behavior, not as exact decompositions of the prediction function.

##### Estimation strategy and model validation

The analysis accounted for the complex sampling structure of the Malawi DHS by incorporating DHS sampling weights directly into the generalized additive model using the argument weights, ensuring that parameter estimation reflected the unequal probability of selection inherent in the DHS survey design. Weighted estimation aligns the fitted model with the population-level distribution of key strata, thereby partially addressing stratification without requiring explicit stratum-specific modeling.

We implemented a multi-component validation strategy to assess the predictive performance and stability of the generalized additive model. First, we conducted 10-fold cross-validation, refitting the model on 90% of the data and evaluating predictions on the held-out fold, rotating across all partitions. Model discrimination was quantified using ROC curves and AUC, while calibration was evaluated through observed-vs.-predicted plots and calibration slopes. To assess robustness to smoothing choices, we performed sensitivity analyses by varying the basis dimension and smoothing parameter selection criteria. Finally, we compared the GAM's predictive performance with that of a simpler weighted logistic regression model to evaluate the added value of nonlinear and spatial smooths.

##### Prediction grid and mapping

Spatial predictions were generated on a regular grid (0.01° resolution) covering the country of Malawi. The grid was clipped to national and district boundaries to avoid extrapolation into unsampled areas, and predictions near the edges of the sampled domain were interpreted cautiously. This procedure yields a high-resolution, spatially coherent surface while ensuring that interpolation remains within meaningful geographic limits.

##### Thin-plate spline implementation

We provide additional detail on the implementation of the thin-plate spline smoothers. Basis dimensions (*k*) were set to balance flexibility and computational feasibility, with higher *k* for the spatial surface and smaller values for univariate smooths. We checked concurvity to ensure smooth terms were not redundant, and smoothing parameters were selected via REML. Model adequacy was evaluated using residual diagnostics and basis-dimension checks (gam.check) to confirm that the chosen bases were sufficient without overfitting.

## Results

### Descriptive characteristics of the study population

Table 1 describes sample characteristics by HIV status. When looking into respondents' age and education, findings show that individuals' characteristics differ by HIV status. For instance, people tested HIV + are older (M=34.8±9.1) compared with HIV- respondents (M=27.7±9.8). Furthermore, people living with HIV are less educated than people without HIV: (6.3±4.1vs.6.5±3.8). Turning to HIV prevalence for categorical variables, findings can be summarised as follows. First, household wealth index, a proxy of socioeconomic status, is associated with HIV status. Indeed, HIV prevalence is higher among better-off households (10.9%) compared with middle (8.7%) and poor households (7.5%). Second, HIV prevalence is higher among females than males (11% vs. 7.2%) and urban residents (15.4% vs. 7.9%).

**Table 1:** Description of the sample by HIV status (weighted).

Variable	Category	HIV−	HIV+
Age (years)	Mean ± SD	27.7 ± 9.8	34.8 ± 9.1
Education (years)	Mean ± SD	6.5 ± 3.8	6.3 ± 4.1
Household wealth, % (n)	Poor	92.5 (4,901)	7.5 (398)
	Middle	91.3 (2,549)	8.7 (243)
	Rich	89.1 (5,567)	10.9 (683)
Prior HIV testing, % (n)	Tested and received results	89.2 (9,748)	10.8 (1,182)
	Tested and did not receive results	87.4 (166)	12.6 (24)
	Never tested	96.3 (3,103)	3.7 (119)
Residence, % (n)	Urban	84.6 (2,167)	15.4 (393)
	Rural	92.1 (10,850)	7.9 (931)
Sex, % (n)	Female	89.0 (6,857)	11.0 (844)
	Male	92.8 (6,159)	7.2 (481)

HIV, Human Immunodeficiency Virus; SD, standard deviation.

### Model performance and smooth terms

The binomial GAM with a logit link showed modest explanatory power, with an adjusted pseudo $R^{2}=0.096$ and 14.4% deviance explained. Spatial smooths over the two-dimensional location surface s(X,Y) were highly significant, indicating substantial geographic structure in HIV risk. Age also exhibited a strong nonlinear association with HIV status, reflected in a significant smooth terms (age) with an estimated 4 effective degrees of freedom (*edf*). In contrast, the smooth for educations (educ) was not statistically significant, suggesting little evidence of a nonlinear effect after accounting for other predictors.

Among the parametric terms, individuals living in rural areas and males had significantly lower odds of HIV infection. Wealth indicators (middle and rich categories) were not associated with HIV status. Prior testing history showed mixed effects: those who had been tested but did not receive results had higher odds of HIV, whereas individuals who had never been tested had significantly lower odds compared to the reference category.

Overall, the model identifies strong spatial and age-related patterns in HIV risk, while socioeconomic gradients appear weaker once smooth terms and covariates are included.

### Spatial patterns of HIV prevalence

#### Preliminary analyses

**Figure 2:**
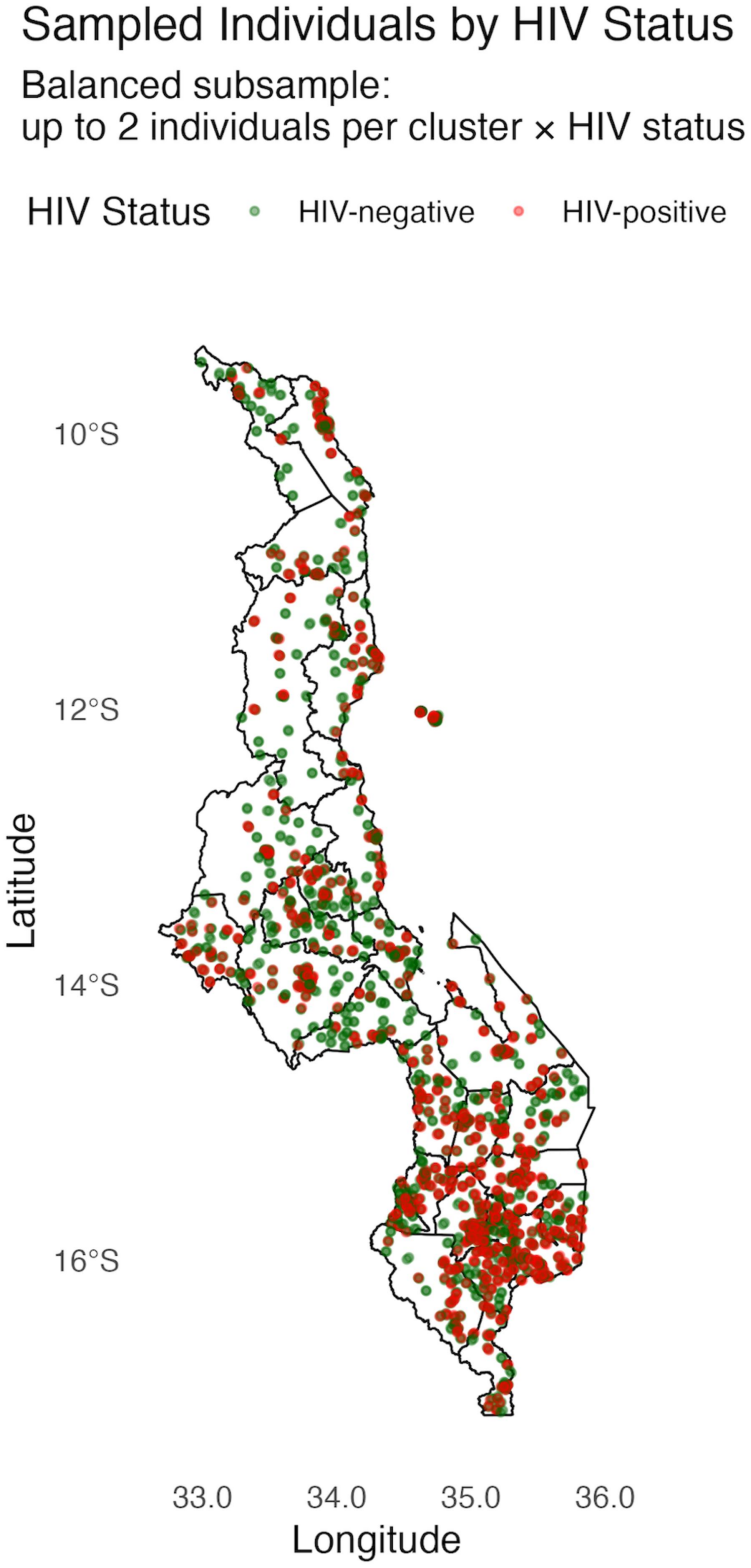
Sampled individuals by HIV status. Maps generated using the R package rgeoboundaries, which accesses the GeoBoundaries database of political administrative boundaries (CC BY 4.0, https://www.geoboundaries.org).

Preliminary analyses consisted of identifying spatial locations of HIV cases vs. controls (seronegative people) to get a sense of geographical disparities. The resulting map was fuzzy due to higher number of sampled individuals. To get an interpretable map, two individuals were randomly sampled by cluster (See Figure 2).

From Figure 2, HIV status exhibits clear geographic variation across the sampled region, with both HIV-positive and HIV-negative individuals distributed unevenly across space. The map shows a balanced subsample (up to 2 individuals per cluster per HIV status), allowing for fair visual comparison. Clusters with HIV-positive individuals (red dots) appear more concentrated in certain areas, suggesting potential spatial hotspots of HIV prevalence. This spatial patterning supports the use of geographic modeling and targeted public health interventions.

### Total sample and sex-specific patterns

#### Smoothed HIV prevalence

**Figure 3:**
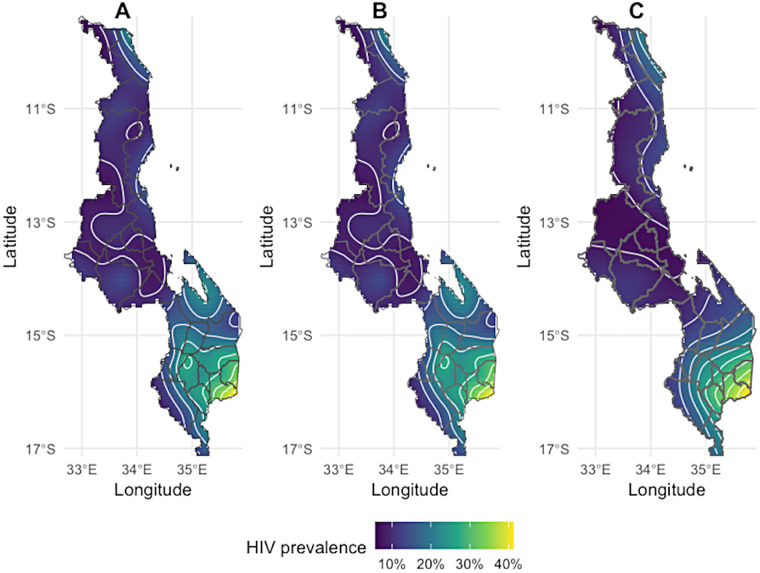
Smoothed HIV prevalence in Malawi. Spatially smoothed estimates of HIV prevalence are shown for **(A)** both sexes combined, **(B)** females, and **(C)** males. Maps generated using the R package rgeoboundaries, which accesses the GeoBoundaries database of political administrative boundaries (CC BY 4.0, https://www.geoboundaries.org).

The three maps (see Figure 3) consistently show strong spatial heterogeneity in HIV prevalence across Malawi, with several robust patterns emerging:

First, there is a pronounced North–South gradient. Across all panels, HIV prevalence is lowest in the Northern Region, moderate in much of the Central Region, and highest in the Southern Region. This gradient remains stable regardless of model specification, indicating a persistent geographic structure of HIV risk.

Second, high-prevalence hotspots are identified in the Southern Region. The southern districts, particularly in the south-eastern and South-Western parts of the country, exhibit the highest predicted prevalence levels (often exceeding 30%–40%). These hotspots are spatially contiguous, suggesting area-level drivers rather than isolated local effects.

Third, there is consistency across panels (A–C). While minor differences in intensity and smoothness appear across panels, the overall spatial pattern is remarkably consistent, implying that the detected hotspots are not artifacts of a single model specification. Additionally, spatial effects explain a substantial share of HIV risk beyond individual-level covariates.

Fourth, findings indicate an observed localized spatial variation within regions. Even within regions of generally low or moderate prevalence, there are pockets of elevated risk, particularly along transport corridors and densely populated areas. This highlights the importance of sub-regional targeting rather than region-wide interventions alone.

Fifth, the persistence of high-prevalence zones after smoothing suggests these spatial patterns may reflect unmeasured contextual or structural factors, including mobility, urbanization, or social networks. Effective HIV control in Malawi therefore requires geographically targeted prevention and treatment strategies, especially in the Southern Region.

#### Uncertainty around HIV prevalence

**Figure 4:**
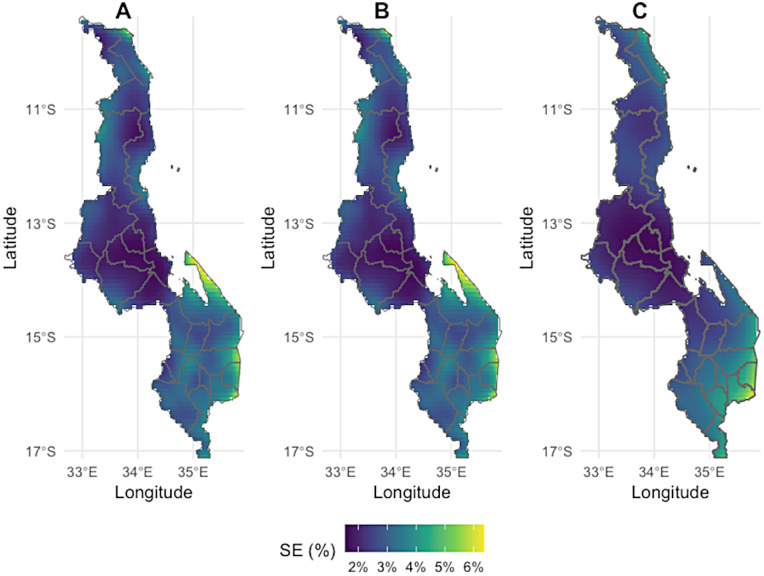
Standard errors for smoothed HIV prevalence estimates in Malawi. Maps show the estimated standard error (SE, %) of HIV prevalence predictions for **(A)** both sexes combined, **(B)** females, and **(C)** males. Maps generated using the R package rgeoboundaries, which accesses the GeoBoundaries database of political administrative boundaries (CC BY 4.0, https://www.geoboundaries.org).

Figure 4 depict the spatial distribution of uncertainty (standard error, SE %) in estimated HIV prevalence across Malawi and reveal several clear patterns:

First, findings showed lower uncertainty in the Northern and much of the Central Region. Across all panels, uncertainty is generally lowest in the Northern Region and large parts of the Central Region, indicating relatively precise prevalence estimates in these areas. This likely reflects adequate sample sizes and more homogeneous HIV risk within these regions.

Second, there was also higher uncertainty concentrated in the Southern Region. The Southern Region shows consistently higher SE values, particularly in the south-eastern and south-western districts, where HIV prevalence is also highest. This pattern suggests greater heterogeneity in risk, possible clustering of cases, and/or smaller effective sample sizes in these areas.

Third, an alignment between high prevalence and high uncertainty Areas with elevated HIV prevalence tend to coincide with higher estimation uncertainty, highlighting that while these locations are clearly high burden, the exact magnitude of prevalence varies more locally. This reinforces the need for cautious interpretation of point estimates in hotspot areas.

Fourth, we observed a consistency across model specifications. Despite minor differences in intensity, the overall spatial pattern of uncertainty remains stable across Panels A–C, suggesting that the uncertainty structure is robust to modeling choices and is driven primarily by data characteristics rather than model instability.

#### Spatial-only predictions of HIV prevalence

**Figure 5:**
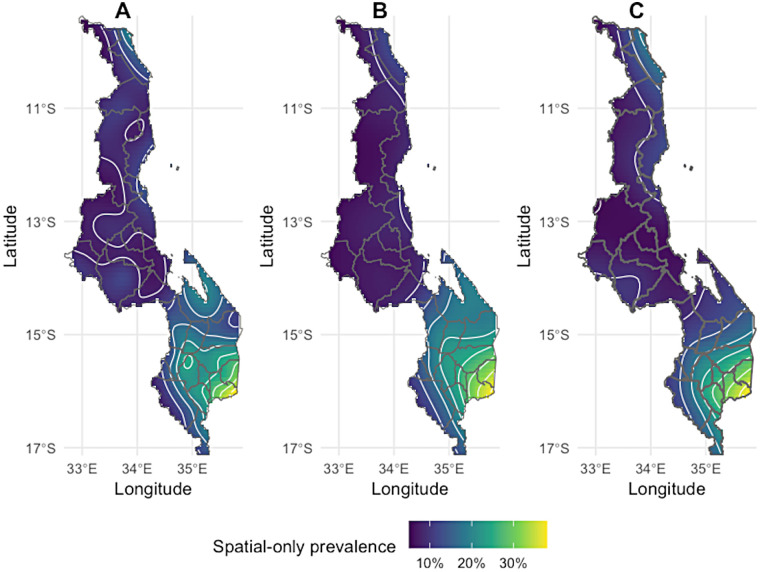
Maps display spatial-only predictions of HIV prevalence for **(A)** both sexes combined, **(B)** females, and **(C)** males, based on geographic smooths without covariate adjustment. Maps generated using the R package rgeoboundaries, which accesses the GeoBoundaries database of political administrative boundaries (CC BY 4.0, https://www.geoboundaries.org).

The maps in Figure 5 show HIV prevalence estimated using spatial effects only, capturing residual geographic structure after excluding individual-level covariates. Several consistent patterns emerge. Findings indicate a strong North–South gradient. Indeed, HIV prevalence is lowest across most of the Northern Region, modest in the Central Region, and highest in the Southern Region, especially the south-eastern and southern lake-shore areas. This confirms a pronounced geographic gradient that persists even without demographic or behavioral predictors.

Additionally, distinct high-prevalence hotspots are visible in southern Malawi, with prevalence exceeding ∼20%–30% in parts of the south-east. These areas remain high burden purely due to spatial location, indicating strong unobserved or contextual drivers (e.g., mobility corridors, urbanization, fishing communities).

Clearly, spatial smoothing reveals coherent regional patterns. The surfaces are smooth and contiguous, suggesting that HIV risk is spatially clustered rather than randomly distributed. Neighboring districts tend to share similar prevalence levels, supporting the use of spatial models.

Finally, findings show consistency across panels. While Panels A–C may differ slightly in magnitude or smoothness, the location of high- and low-prevalence zones is stable, indicating robust spatial signal and limited sensitivity to model specification.

The spatial-only HIV prevalence maps reveal a strong and stable geographic pattern in Malawi, with concentrated high prevalence in the Southern Region and low prevalence in the North, underscoring the importance of location-specific HIV dynamics even before accounting for individual-level risk factors.

#### Shapley decomposition of HIV risk

**Figure 6:**
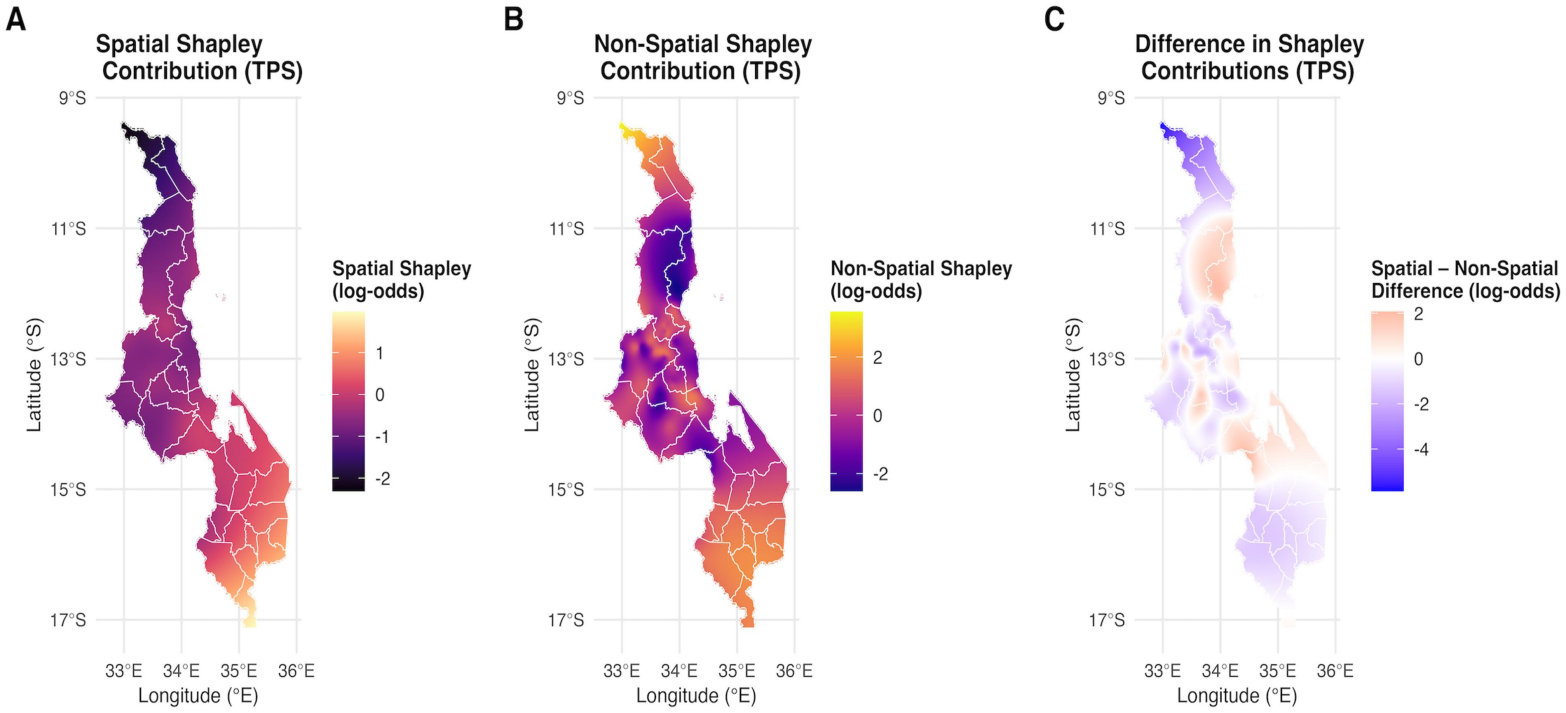
Shapley contributions to HIV risk predictions in Malawi. Panel **(A)** shows the spatial Shapley contribution derived from thin plate spline (TPS) smooths, highlighting geographic variation in predicted HIV risk attributable to location alone. Panel **(B)** displays the non-spatial Shapley contribution, reflecting the influence of individual-level covariates. Panel **(C)** presents the difference between spatial and non-spatial contributions, revealing regions where geographic context amplifies or attenuates the effects of non-spatial predictors. Maps generated using the R package rgeoboundaries, which accesses the GeoBoundaries database of political administrative boundaries (CC BY 4.0, https://www.geoboundaries.org).

To disentangle the relative influence of geographic context and individual-level characteristics on HIV risk, we applied Shapley decomposition to the fitted binomial GAM. Figure 6 presents three maps summarizing these contributions across Malawi. Panel A displays the spatial Shapley contribution, quantifying the effect of location alone on HIV risk. Regions with higher spatial values—particularly in the southeast—indicate areas where geographic context strongly elevates predicted risk, independent of covariates. Panel B shows the non-spatial Shapley contribution, reflecting the influence of individual-level predictors such as age, education, and behavioral factors. Panel C illustrates the difference between spatial and non-spatial contributions (Spatial—Non-Spatial), revealing zones where geographic context amplifies or attenuates the effects of personal characteristics. Together, these maps highlight the spatial heterogeneity of HIV risk and underscore the importance of accounting for both geographic and demographic factors in epidemiological modeling and intervention planning.

#### What are the most important factors associated with HIV risk in Malawi: spatial vs. sociodemographics

Previous studies have mainly focused on sociodemographics to explain HIV prevalence in developing countries. Therefore, and taking advantages of Shapley decomposition of HIV risk in the country, the paper disentangled the contribution of space and sociodemographics, which a critical to devise more effective HIV interventions. Findings indicate that measured individual-level covariates explained more variation in predicted HIV risk than residual spatial structure in most districts in Malawi (See Table 2).

**Table 2:** Districts where spatial effects contribute more than covariate effects (spatial—covariate >0).

District	Mean Difference
Nkhata Bay	1.260
Dedza	0.745
Mangochi	0.559
Ntcheu	0.239
Mzimba	0.194

These include Mzimba and Nkhata Bay (Northern region); Dedza and Ntechu (Central region); Mangochi (Southern region). Overall, these findings confirm that HIV risk in sub-Saharan Africa and Malawi is still driven by people's sociodemographics.

## Discussion

### Main findings

This study provides a detailed spatial characterization of HIV prevalence in Malawi using a generalized additive modeling (GAM) framework that integrates individual-level survey data with flexible spatial smoothers. By leveraging the strengths of GAMs—nonlinear functional forms, spatial smoothing, and partial pooling across sparsely sampled areas—models generated stable, high-resolution prevalence surfaces that reveal substantial geographic heterogeneity in HIV burden across the country.

Our findings corroborate previous studies (7, 12, 23) and highlight persistent spatial disparities in HIV prevalence, with the highest predicted burden concentrated in the southern region, particularly in districts surrounding Blantyre, Zomba, and the Shire Highlands. These patterns are consistent with historical epidemiological trends and reflect the influence of urbanization, mobility, and socioeconomic gradients on HIV transmission dynamics (10). In contrast, the Northern region exhibited lower and more spatially homogeneous prevalence, suggesting different structural and behavioral drivers of the epidemic in the country (12, 24). For instance, previous studies showed that marital status, education, and age are strong drivers of the spread of HIV infection in Malawi (25). Therefore, although the paper advanced our understanding of the spread of HIV infection in the country, sociodemographics are equally important to controlling the epidemic. Indeed, findings indicated that space was an important driver of HIV in 18% of the districts.

The sex-specific analyses further underscore the importance of disaggregated modeling: females exhibited more pronounced and spatially concentrated hotspots, while male prevalence patterns were more diffuse. These differences align with known gender disparities in HIV acquisition risk, testing behavior, and access to prevention services (26, 27). Indeed, previous studies documented gender disparities in HIV acquisition risk (28), testing behavior (29), and access to prevention services in Malawi, including earlier testing and care engagement among women and delayed diagnosis among men (10, 26, 30).

The maps demonstrate a clear and persistent spatial pattern of HIV prevalence in Malawi, characterized by a pronounced concentration of risk in the southern region. Although prevalence estimates remain spatially stable across the country, the associated uncertainty is unevenly distributed and highest in these same high-burden southern districts, underscoring the need for geographically targeted data-strengthening efforts alongside focused HIV control strategies. Because these estimates reflect spatial structure alone, they capture baseline geographic risk and highlight persistent hotspots where place-based factors exert influence beyond individual characteristics (12, 23, 31). Together, these spatial patterns provide valuable guidance for geographically targeted HIV interventions, surveillance prioritization, and the generation of hypotheses about contextual drivers of transmission.

The use of effect-coded categorical variables improved model interpretability by centering covariate effects around the grand mean rather than a single reference category. This approach also enhanced numerical stability, particularly in the presence of interactions between demographic factors and spatial structure. The spatial smoother captured broad geographic gradients while allowing for localized deviations, producing a continuous risk surface that avoids the instability of direct survey estimates. This is particularly important in districts with small sample sizes, where raw prevalence estimates can be highly unstable (23, 32, 33).

Although the model incorporated several well-established predictors of HIV infection, we acknowledge that important determinants—such as marital status, sexual behavior, sexually transmitted infection history, migration, and circumcision—were not included. The decision to exclude these variables reflects both the computational constraints of fitting high-dimensional spatial GAMs with large survey datasets and the need to maintain a parsimonious specification that avoids overfitting when estimating complex thin-plate spline surfaces. Omitting these factors may contribute to residual spatial confounding, whereby unmeasured behavioral or clinical determinants manifest as spatial structure in the estimated surface. As a result, the spatial smooth may partially absorb variation attributable to unobserved individual-level risk factors, potentially inflating the magnitude of spatial effects. We therefore interpret the spatial component as capturing broad-scale geographic patterns in HIV risk rather than causal spatial processes, and we emphasize that future work incorporating richer behavioral and clinical covariates could help further disentangle spatial and non-spatial sources of heterogeneity.

From a policy perspective, the spatially refined prevalence estimates generated by this model offer actionable insights for HIV program planning. High-burden districts identified represent priority areas for intensified testing, prevention, and treatment interventions. The ability to quantify uncertainty at each prediction location provides an additional layer of decision support, enabling policymakers to distinguish between areas of genuinely elevated risk and areas where additional data collection may be warranted. These results can inform differentiated service delivery models, optimize resource allocation, and support Malawi's progress toward achieving and sustaining the UNAIDS 95–95–95 targets (32, 34).

This study also demonstrates the utility of GAM as a middle ground between fully parametric regression models and more computationally intensive Bayesian hierarchical spatial models (35–37). Although we do not specify a formal spatial dependence model, the thin-plate spline smoother effectively represents large-scale geographic gradients and produces coherent, smooth prevalence surfaces. Future work could extend this framework by incorporating temporal data to model changes in HIV prevalence over time, integrating mobility or network data, or comparing GAM-based estimates with those from Bayesian geostatistical models.

Overall, this paper provides a robust and flexible framework for sub-national HIV estimation in Malawi. By combining rigorous statistical modeling with high-resolution spatial prediction, it contributes to a deeper understanding of HIV epidemiology and supports evidence-based strategies to accelerate progress toward epidemic control. Despite these efforts to further our understanding of the spatial contribution in spreading HIV infection, it still is driven by sociodemographic factors such as age and education, among others.

### Limitations

This study has several limitations that should be considered when interpreting the findings. First, the analysis relies on cross-sectional DHS data, which limits the ability to infer causal relationships or temporal trends in HIV prevalence. Although the generalized additive model captures nonlinear and spatial structure, it cannot account for changes in HIV epidemiology over time or for dynamic processes such as migration, treatment scale-up, or behavioral shifts. Second, DHS cluster coordinates are intentionally displaced to protect participant confidentiality, which introduces spatial uncertainty. While the spatial smoother mitigates some of this noise, fine-scale geographic patterns may be attenuated. Third, several covariates—such as sexual behavior, Sexually Transmitted Infections (STI) symptoms, and prior HIV testing—are self-reported and subject to recall or social desirability bias. Fourth, although GAMs provide flexible modeling of spatial and nonlinear effects, they do not explicitly incorporate spatial dependence through formal geostatistical priors. As a result, uncertainty estimates may be more conservative than those produced by fully Bayesian hierarchical spatial models. Finally, the prediction grid assumes smooth variation across space and does not incorporate structural determinants such as mobility networks, health facility distribution, or socioeconomic gradients, which may influence HIV transmission dynamics.

Despite these limitations, the spatial GAM framework offers a robust and interpretable approach for generating sub-national HIV estimates and provides valuable insights for program planning and resource allocation.

## Conclusion

This study provides a comprehensive spatial assessment of HIV prevalence in Malawi using a generalized additive modeling framework that integrates individual-level survey data with flexible spatial smoothers. By leveraging the strengths of GAMs—nonlinear functional forms, spatial smoothing, and partial pooling across sparsely sampled areas—we generated stable, high-resolution prevalence surfaces that reveal substantial geographic heterogeneity in HIV burden.

The results highlight persistent spatial disparities, with the highest predicted prevalence concentrated in southern Malawi and more diffuse patterns in the central and northern regions. Sex-specific analyses further underscore the importance of disaggregated modeling, revealing distinct spatial structures for males and females. These findings align with known epidemiological patterns and emphasize the need for geographically targeted HIV prevention, testing, and treatment strategies.

The spatial GAM approach offers a practical and interpretable alternative to more computationally intensive geostatistical models, while still providing robust uncertainty quantification and smooth sub-national estimates. By producing continuous risk surfaces and district-level summaries, this work supports evidence-based decision-making and can inform resource allocation, differentiated service delivery, and surveillance priorities. As Malawi continues to advance toward the UNAIDS 95–95–95 targets, spatially refined estimates such as these will remain essential for sustaining progress and addressing remaining pockets of elevated HIV burden.

Future work could extend this framework by incorporating temporal data, mobility patterns, or additional structural determinants of HIV risk. Nonetheless, the present analysis demonstrates the value of spatial GAMs for understanding and responding to HIV epidemics in settings where geographic heterogeneity plays a central role.

## Data Availability

Publicly available datasets were analyzed in this study. This data can be found here: https://dhsprogram.com/data/available-datasets.cfm.
