# Supplementary material for: Spatial modeling of HIV prevalence in Malawi using generalized additive models
Source: Front Epidemiol. 2026 Jul 8;6:1867778. doi: 10.3389/fepid.2026.1867778 (PMC13388554; doi:10.3389/fepid.2026.1867778)
Supplement: Supplementary file 1 [file Supplementaryfile1.docx]

**Appendix**

**A.1 Spatial Generalized Additive Model**

Let $Y_{i}\in\{0,1\}$ denote the HIV serostatus of individual $i$, where $Y_{i}=1$ indicates HIV positive.

We assume: $Y_{i}\sim\text{Bernoulli}\left( p_{i} \right)$

The probability of HIV infection is modeled using a logistic generalized additive model (GAM): $\text{logit}\left( p_{i} \right)=\eta_{i}$

$$\eta_{i}=\beta_{0}+f_{s}\left( X_{i},Y_{i} \right)+\beta_{1}\text{Urban}_{i}+f_{a}\left( \text{Age}_{i} \right)+f_{e}\left( \text{Educ}_{i} \right)+\beta_{2}\text{Sex}_{i}+\beta_{3}\text{HWI}_{i}+\beta_{4}\text{TestPrior}_{i}$$

where:

- $f_{s}\left( X_{i},Y_{i} \right)$ is a bivariate thin-plate spline capturing spatial variation,
- $f_{a}\left( \cdot\right)$ and $f_{e}\left( \cdot\right)$ are smooth functions of age and education,
- $\beta's$ denote parametric effects.

The model is estimated using penalized likelihood with restricted maximum likelihood (REML) [[1](#ref-HastieTibshirani1990)–[4](#ref-Wood2017)].

**A.2 Prediction Scale: From Linear Predictor to Probability**

In generalized additive models (GAMs) with a binary outcome, the model estimates a linear predictor:

$$\hat{\eta}_{i}=\mathbf{x}_{i}^{\top}\hat{\boldsymbol{\beta}}+\sum_{j} \hat{f}_{j}\left( z_{ij} \right)$$

This is on the logit scale, meaning it models the log-odds of the outcome:

$$\hat{\eta}_{i}=log\left( \frac{\hat{p}_{i}}{1-\hat{p}_{i}} \right)$$

To interpret predictions in terms of probability, we apply the inverse logit transformation:

$$\hat{p}_{i}=Pr\left( Y_{i}=1\mid\mathbf{x}_{i} \right)=\frac{\exp\left( \hat{\eta}_{i} \right)}{1+exp\left( \hat{\eta}_{i} \right)}$$

This maps any real-valued linear predictor ($\hat{\eta}_{i}\mathbb{\in R}$) to a probability ($\hat{p}_{i}\in\left( 0,1 \right)$) [[1](#ref-HastieTibshirani1990),[4](#ref-Wood2017)].

- **Interpretability**: Probabilities are easier to interpret than log-odds. For example, ($\hat{p}_{i}=0.82$) means an 82% chance of testing positive or receiving ART.
- **Visualization**: Maps and plots of predicted probabilities are more intuitive than log-odds surfaces.
- **Policy relevance**: Thresholds (e.g., $\hat{p}_{i}>0.9$) can guide **targeting** or **resource allocation**.

**A.2.1 Gradient of the predicted probability**

For a binary-outcome GAM with logit link, the predicted probability for unit $i$ is

$$\hat{p}_{i}=Pr\left( Y_{i}=1\mid\mathbf{x}_{i} \right)=\frac{\exp\left( \hat{\eta}_{i} \right)}{1+exp\left( \hat{\eta}_{i} \right)},$$

where $\hat{\eta}_{i}$ is the fitted linear predictor. Differentiating with respect to $\hat{\eta}_{i}$,

$$\frac{\partial\hat{p}_{i}}{\partial\hat{\eta}_{i}}=\hat{p}_{i}\left( 1-\hat{p}_{i} \right).$$

If the linear predictor is

$$\hat{\eta}_{i}=\mathbf{x}_{i}^{\top}\hat{\boldsymbol{\beta}}+\sum_{j} \hat{f}_{j}\left( z_{ij} \right),$$

then, for the parametric covariates,

$$\frac{\partial\hat{\eta}_{i}}{\partial\mathbf{x}_{i}}=\hat{\boldsymbol{\beta}},$$

so by the chain rule

$$\frac{\partial\hat{p}_{i}}{\partial\mathbf{x}_{i}}=\hat{p}_{i}\left( 1-\hat{p}_{i} \right) \hat{\boldsymbol{\beta}}.$$

Componentwise, for covariate $x_{ik}$,

$$\frac{\partial\hat{p}_{i}}{\partial x_{ik}}=\hat{p}_{i}\left( 1-\hat{p}_{i} \right) \hat{\beta}_{k}.$$

**A.3 Shapley Value Decomposition**

To quantify how each predictor contributes to an individual’s predicted HIV probability, we use the Shapley value framework from cooperative game theory [[5](#ref-Shapley1953),[6](#ref-LundbergLee2017)]. In this setting, each predictor is treated as a “player,” and the model prediction is the “payout” that results from different combinations of predictors working together.

Let $\mathbf{x}_{i}=\left( x_{i1},\ldots,x_{ip} \right)$ denote the vector of predictors for individual $i$, and let $\hat{f}\left( \mathbf{x}_{i} \right)=\hat{p}_{i}$ be the predicted HIV probability from the fitted model.

**A.3.1 Definition**

The Shapley value for feature $j$ represents the average marginal contribution of that feature to the prediction, computed across all possible subsets of the remaining predictors [[5](#ref-Shapley1953),[6](#ref-LundbergLee2017)]. Formally:

$$\phi_{ij}=\sum_{S\mathcal{\subseteq F\backslash\{}j\}} \frac{\left| S \right|!\left( p-\left| S \right|-1 \right)!}{p!}\left[ \hat{f}\left( S\cup\{j\} \right)-\hat{f}\left( S \right) \right]$$

where:

- $\mathcal{F}$ is the full set of predictors,
- $S$ is any subset of predictors not containing $j$,
- $\hat{f}\left( S \right)$ is the expected model prediction when only predictors in $S$ are known and all others are marginalized over their empirical distribution.

Intuitively, $\hat{f}\left( S \right)$ represents the model’s prediction if we “turn off” all predictors not in $S$, averaging over their observed variability. The term $\hat{f}\left( S\cup\{j\} \right)-\hat{f}\left( S \right)$ is the incremental contribution of predictor $j$ when added to subset $S$.
The factorial weighting $\frac{\left| S \right|!\left( p-\left| S \right|-1 \right)!}{p!}$ ensures that each ordering of predictors is weighted equally, making the Shapley value the fair average contribution across all possible predictor orderings.

**A.3.2 Properties**

The Shapley decomposition satisfies four desirable axioms [[5](#ref-Shapley1953),[7](#ref-FryerStrumke2021)]:

**1. Efficiency (Completeness)**

This property ensures that the Shapley decomposition fully accounts for an individual’s predicted risk. Specifically, $\sum_{j=1}^{p} \phi_{ij}=\hat{p}_{i}\mathbb{-E}\left[ \hat{p} \right]$ meaning that the sum of all feature‑specific contributions for individual $i$ exactly reproduces the deviation of their predicted probability from the overall mean prediction. Each predictor contributes a positive or negative adjustment, and these adjustments collectively explain why an individual’s estimated HIV risk is higher or lower than the population baseline. This conservation‑of‑prediction principle guarantees that no contribution is omitted or double‑counted, making the decomposition internally coherent and interpretable. The contributions fully explain the deviation of the individual’s prediction from the average prediction.

**2. Symmetry**

The symmetry property ensures that predictors are treated equitably within the Shapley framework. If two predictors contribute identically to the prediction for every possible subset of features—that is, adding either predictor to any subset results in the same change in the model output—then they must receive identical Shapley values. This guarantees that the decomposition does not arbitrarily favor one predictor over another when their marginal contributions are indistinguishable. In practice, symmetry reinforces the fairness of the attribution: predictors that play equivalent roles in shaping an individual’s estimated HIV risk are assigned equal importance in the final explanation.

**3. Dummy (Null) Property**

If a predictor has no effect on the prediction—meaning
$\hat{f}\left( S\cup\{j\} \right)=\hat{f}\left( S \right)$ for all $S$–then $\phi_{ij}=0$.

**4. Additivity**

For two models $f$ and $g$, the Shapley values of the combined model $f+g$ equal the sum of the Shapley values from each model separately.

**A.3.3 Interpretation in the HIV Modeling Context**

In this analysis, **Shapley values** quantify how much each predictor—such as age, sex, behavioral factors, or spatial random effects—pushes an individual’s **predicted HIV probability above or below the national average** [[6](#ref-LundbergLee2017),[8](#ref-AasJullumLoland2021)]. Because the decomposition is grounded in cooperative game theory, it provides a principled and fair way to compare contributions across heterogeneous predictors, including spatial effects.

**A.4 Shapley Values in a GAM Context**

Although the GAM is additive on the link scale, **Shapley values** are computed on the **prediction scale**, accounting for nonlinearities [[4](#ref-Wood2017),[6](#ref-LundbergLee2017)]:

$$\hat{p}_{i}=g^{-1}\left( \beta_{0}+f_{s}\left( X_{i},Y_{i} \right)+\sum_{k} f_{k}\left( x_{ik} \right) \right)$$

Shapley values therefore represent **local marginal contributions** of predictors to predicted HIV probability, not coefficients or partial derivatives.

**A.5 Spatial Shapley Contribution**

The spatial smooth $f_{s}\left( X,Y \right)$ is represented in the Shapley framework by the joint contribution of coordinates $\left( X,Y \right)$.

For individual $i$, the **spatial Shapley contribution** is defined as:

$$\phi_{i}^{\text{spatial}}=\phi_{iX}+\phi_{iY}$$

This quantity captures the contribution of **unmeasured spatial context**, including environmental, social, and epidemiological processes not explicitly modeled [[9](#ref-DiggleTawnMoyeed1998),[10](#ref-Lawson2018)].

**A.6 Covariate-Driven Contribution**

The **covariate-driven Shapley contribution** is defined as:

$$\phi_{i}^{\text{covariate}}=\sum_{j\mathcal{\in F\backslash\{}X,Y\}} \phi_{ij}$$

This represents the portion of predicted HIV risk attributable to observed individual and household characteristics.

**A.7 Interpretation of Shapley Contributions**

For each individual $i$:

$$\hat{p}_{i}\mathbb{=E}\left[ \hat{p} \right]+\phi_{i}^{\text{spatial}}+\phi_{i}^{\text{covariate}}$$

- $\mathbb{E}\left[ \hat{p} \right]$ : The average predicted probability across all individuals in the dataset. This serves as a baseline or overall mean prediction before considering any individual-specific effects.
- $\phi_{i}^{\text{spatial}}$ : The Shapley contribution from spatial effects for individual $i$. This quantifies how much the individual’s location or spatial context influences their predicted probability, isolating the effect of geography or spatial variation.
- $\phi_{i}^{\text{covariate}}$: The Shapley contribution from covariate effects for individual $i$. This captures the influence of the individual’s specific covariate values (such as age, sex, socioeconomic status, etc.) on their predicted probability.
- This decomposition is useful because it breaks down the prediction into interpretable parts, attributing how much of the predicted probability comes from the overall average, spatial factors, and individual covariates. It helps understand the relative importance and contribution of spatial versus non-spatial predictors for each individual prediction. This can be especially valuable for interpreting complex models like spatial GAMs, where effects are not always straightforward.

**A.7 Shapley Contributions: Spatial vers Covariates dominance**

- $\phi_{i}^{\text{spatial}}>0$: spatial context increases predicted HIV risk
- $\phi_{i}^{\text{spatial}}<0$: spatial context decreases predicted HIV risk
- $\phi_{i}^{\text{covariate}}>0$: observed covariates increase predicted HIV risk

These contributions explain **model predictions**, not causal effects.

**A.8 Methodological Limitations**

First, **Shapley values** depend on the empirical distribution of the predictors used in the estimations. Second, correlated covariates may share contributions in the estimations, making the interpretation fuzzy. Third, spatial Shapley values reflect residual spatial structure, not specific mechanisms. Fourth, **computational complexity** grows exponentially with the number of predictors included in the analyses [[7](#ref-FryerStrumke2021),[8](#ref-AasJullumLoland2021)]. Therefore, the paper included a limited number of covariates.

**References**

1. Hastie, T. J. & Tibshirani, R. J. *Generalized Additive Models*. (Chapman; Hall, London, 1990).

2. Wood, S. N. Thin plate regression splines. *Journal of the Royal Statistical Society: Series B (Statistical Methodology)* **65**, 95–114 (2003).

3. Wood, S. N. Fast stable restricted maximum likelihood and marginal likelihood estimation of semiparametric generalized linear models. *Journal of the Royal Statistical Society: Series B (Statistical Methodology)* **73**, 3–36 (2011).

4. Wood, S. N. *Generalized Additive Models: An Introduction with r*. (Chapman; Hall/CRC, Boca Raton, FL, 2017).

5. Shapley, L. S. A value for n-person games. 307–317 (1953).

6. Lundberg, S. M. & Lee, S.-I. A unified approach to interpreting model predictions. *Advances in Neural Information Processing Systems* **30**, 4765–4774 (2017).

7. Fryer, D. & Strumke, I. Shapley values for feature selection: The good, the bad, and the axioms. *IEEE Access* **9**, 144352–144360 (2021).

8. Aas, K., Jullum, M. & L land, A. Explaining individual predictions when features are dependent: More accurate approximations to Shapley values. *Artificial Intelligence* **298**, 103502 (2021).

9. Diggle, P. J., Tawn, J. A. & Moyeed, R. A. Model-based geostatistics. *Journal of the Royal Statistical Society: Series C (Applied Statistics)* **47**, 299–350 (1998).

10. Lawson, A. B. *Bayesian Disease Mapping: Hierarchical Modeling in Spatial Epidemiology*. (Chapman; Hall/CRC, Boca Raton, FL, 2018).
